# Increased Perfusion in Normal Appearing White Matter in High Inflammatory Multiple Sclerosis Patients

**DOI:** 10.1371/journal.pone.0119356

**Published:** 2015-03-16

**Authors:** Maxim Bester, Nils Daniel Forkert, Jan Patrick Stellmann, Lilian Aly, Anna Drabik, Kim Lea Young, Christoph Heesen, Jens Fiehler, Susanne Siemonsen

**Affiliations:** 1 Department of Diagnostic and Interventional Neuroradiology, University Medical Centre Hamburg-Eppendorf, Hamburg, Germany; 2 Department of Radiology and Hotchkiss Brain Institute, University of Calgary, Calgary, Canada; 3 Department of Neurology, Institute for Neuroimmunology and Clinical MS Research, University Medical Centre Hamburg-Eppendorf, Hamburg, Germany; 4 Department of Neurology, Klinikum rechts der Isar, Technische Universität München, Munich, Germany; 5 Department of Medical Biometry and Epidemiology, University Medical Centre Hamburg-Eppendorf, Hamburg, Germany; Brighton and Sussex Medical School, UNITED KINGDOM

## Abstract

**Purpose:**

Although cerebral perfusion alterations have long been acknowledged in multiple sclerosis (MS), the relationship between measurable perfusion changes and the status of highly active MS has not been examined. We hypothesized that alteration of perfusion can be detected in normal appearing white matter and is increased in high inflammatory patients.

**Materials and Methods:**

Thirty-three patients with relapsing-remitting MS underwent four monthly 3T MRI scans including dynamic susceptibility contrast perfusion-weighted MRI. Cerebral blood flow (CBF) and cerebral blood volume (CBV) were measured in normal appearing white matter. Patients were stratified in a high- and low-inflammatory group according to the number of new contrast enhancing lesions.

**Results:**

Thirteen patients were classified as high-inflammatory. Compared to low-inflammatory patients, the high-inflammatory group demonstrated significantly higher CBV (p = 0.001) and CBF (p = 0.014) values. A mixed model analysis to assess independent variables associated with CBV and CBF revealed that white matter lesion load and atrophy measurements had no significant influence on CBF and CBV.

**Conclusion:**

This work provides evidence that high inflammatory lesion load is associated with increased CBV and CBF, underlining the role of global modified microcirculation prior to leakage of the blood-brain barrier in the pathophysiology of MS. Perfusion changes might therefore be sensitive to active inflammation apart from lesion development without local blood–brain barrier breakdown, and could be utilized to further assess the metabolic aspect of current inflammation.

## Introduction

Multiple sclerosis (MS) is a chronic demyelinating disease of the central nervous system and white matter (WM) lesions are visualized using T2w magnetic resonance imaging (MRI) in most patients. However, weak correlation of T2 lesion volume (T2-LV) with clinical impairment [1] has stimulated efforts to identify quantitative imaging measures to better monitor pathological processes of MS with relevance for clinical presentation. While contrast enhancement of the lesions on T1w scans is the most striking aspect of acute inflammation in MS, it is increasingly recognized that a more widespread and subtle form of inflammation occurs within the normal-appearing white matter (NAWM) [2,3].

Since the earliest descriptions of MS, the affinity of inflammatory cells and acute demyelinating lesions to blood vessels has been described [4] but until recently did not receive much attention. Previous neuropathological studies, demonstrating perivascular inflammatory changes and hyalinization in venous vessel walls in NAWM of patients with MS, have raised the question if a vascular disease mechanism may underlie MS pathology [5,6]. Studying ongoing inflammatory processes in NAWM by use of structural MRI has been challenging. These processes are not associated with a clear break-down of the blood–brain barrier (BBB) which can be detected as regions of gadolinium contrast enhancement in highly inflammatory lesions [7]. Previous imaging studies have used perfusion-weighted MRI (PWI) techniques to measure cerebral perfusion in MS patients. These studies have identified both decrease [8–10] and increase [11,12] of perfusion in WM. Studies focusing on lesion formation could detect focal increased blood flow up to 6 months prior to lesion formation [13] and increased cerebral blood flow (CBF) and cerebral blood volume (CBV) in contrast enhancing lesions [14]. Inflammation with increased tissue metabolic demand has been proposed as one mechanism of increased WM and lesion perfusion. However, the relationship between measurable perfusion alteration and visible inflammation in structural MRI has not been examined so far.

We assume that assessment of the association between the extent of gadolinium enhancing lesions and perfusion abnormalities in NAWM might give important information about the relationship between focal inflammation and global hemodynamic changes. The question arises whether PWI might detect an increase of CBV and CBF in WM in high inflammatory MS patients that could indicate a potentially reversible state of inflammation-related vasodilatation [13] accompanying the formation of inflammatory lesions.

In this study, we focused on the inflammatory aspect of MS and investigate a high inflammatory patient cohort with dynamic susceptibility contrast perfusion-weighted MR imaging (DSC-PWI). We hypothesized that patients with a high number of contrast enhancing lesions have increased NAWM perfusion situation as measured by CBV and CBF.

The aims of our study were: a) to measure the tissue perfusion in NAWM of high inflammatory MS patients using DSC-PWI; b) to investigate the relationship between perfusion alteration in NAWM and measures of WM lesion and brain volume; and c) to explore the relationship between perfusion parameters derived from DSC-PWI and clinical impairment.

## Methods

### Subjects

Thirty-three patients meeting McDonald diagnostic criteria for MS [15] with relapsing-remitting disease course (RR-MS) were recruited prospectively from the in house MS day-unit when meeting all inclusion criteria. All patients were steroid-treatment and disease modifying drugs free for at least three months. Exclusion criteria were the presence of other relevant diseases and contraindications to perform MRI. Patients underwent four MRI examinations (MRI-1 to MRI-4) on monthly basis (evaluable DSC-MRI in all four timepoints were acquired in 25 participants [75.7%], in 8 patients one DSC-MRI dataset had to be excluded from the analysis due to strong movement of the patient). Patients were separated into a high inflammatory (HI) and a low inflammatory (LI) groups according to the number of newly emerged contrast enhancing lesions (nGd-L) retrospectively after the last MRI. If a patient developed at least one new contrast enhancing lesion in at least three (out of the four) MRI scans, the patient was stratified into the HI group. Although a universally accepted definition for highly active MS does not exist, recent studies make use of the accumulation of contrast enhancing lesions to define high disease activity [16]. Disability was assessed using the expanded disability status scale (EDSS) [17]. Approval for this study was obtained from Hamburg medical association ethical committee and written informed consent was received from all subjects.

### MR Imaging acquisition

MRI was performed using a 3T scanner (Skyra, Siemens Medical Systems, Erlangen, Germany) with a 32 channel phased-array head coil using following protocol: a) axial T2/PDw turbo spin dual echo (TSE) sequence (repetition time [TR] = 2800 ms, echo time [TE] = 18/90 ms, field-of-view [FOV] = 240×180 mm^2^, matrix = 256×256, voxel size = 0.5×0.5×3 mm^3^, 43 contiguous slices); b) sagittal 3D magnetization prepared rapid acquisition of gradient echoes sequence (MPRAGE) (TR = 1900 ms, inversion time [TI] = 900 ms, TE = 2.46 ms, flip-angle α = 9°, FOV = 240×240 mm^2^, matrix = 256×256, 192 slices, voxel size = 0.9×0.9×0.9 mm^3^); c) sagittal 3D fluid attenuated inversion recovery sequence (FLAIR) (TR = 4700 ms, TI = 1800 ms, TE = 392 ms, FOV = 240×240 mm^2^, matrix = 320×320, 192 slices, voxel size = 0.8×0.8×0.9 mm^3^); d) axial EPI (echo planar imaging) DSC-PWI (TR = 1920 ms, TE = 2.46 ms, flip-angle α = 900°, FOV = 240×240 mm^2^, matrix = 128×128, voxel size = 1.9×1.9×4 mm^3^, 27 contiguous slices). Ten milliliters of gadoterate dimeglumine (Dotarem, Guerbet, France) (1 mmol/mL) were administered by an intravenous pump injector at a rate of 5mL/s followed by a 25 mL bolus of saline. A total of 50 DSC images were acquired with the contrast injection starting at the 10th image; e) post-contrast MPRAGE (see b) acquired 8 minutes after intravenous injection of Dotarem.

### Lesion volume measurements

All data processing was performed by an observer blinded to the subjects' identity.

T2-LV, T1-hypointense lesion volume (T1-LV) and contrast enhancing lesion volume (Gd-L) measurements were performed by a trained physician using a semiautomatic segmentation technique based on local thresholding using Analyze (v11, Mayo Clinic Biomedical Imaging, Rochester, MN, USA). The delineated lesions were used for lesion volume determination and saved as binary lesion maps for further processing. The number of nGd-L and Gd-L was counted for each subject and four time points on post-contrast MPRAGE images.

### Volume measurements

Normalized values of gray matter volume (GMV) and white matter volume (WMV) were measured in the pre-contrast MPRAGE datasets using SIENAX [18]. SIENAX automatically calculates the brain volume and applies a normalization factor to correct for skull size. To correct for misclassification of GMV in presence of T1w hypointense lesions, each segmented lesion was filled with the mean intensity of the NAWM present in the same slice [19].

### DSC-Perfusion

The processing of the DSC-PWI datasets was performed using the in-house developed software AnToNIa [20]. Briefly described, the processing steps for perfusion parameter map calculation performed in AnToNIa includes a motion correction, conversion of the signal curves to absolute contrast agent concentration time curves, slice-time correction and calculation of the four perfusion parameter maps (CBV = cerebral blood volume, CBF = cerebral blood flow, MTT = mean transit time, TTP = time-to-peak) using the local-density random walk (LDRW) model [21]. Further data postprocessing was performed using the FSL Software library (v5.0, FMRIB, Oxford, UK, http://www.fmrib.ox.ac.uk/fsl). Registration between DSC-PWI data (and consequently the CBV/CBF maps) and T2w images was performed in a combined boundary based linear registration and non-linear transformation [22] to correct for distortion due to echo-planar imaging artifacts. The 4^th^ PWI acquisition was used as the reference for the registration providing the best T2 contrast. The T1w scan and consequently the GM/WM masks were transformed to the T2 space by affine registration.

**Fig 1:**
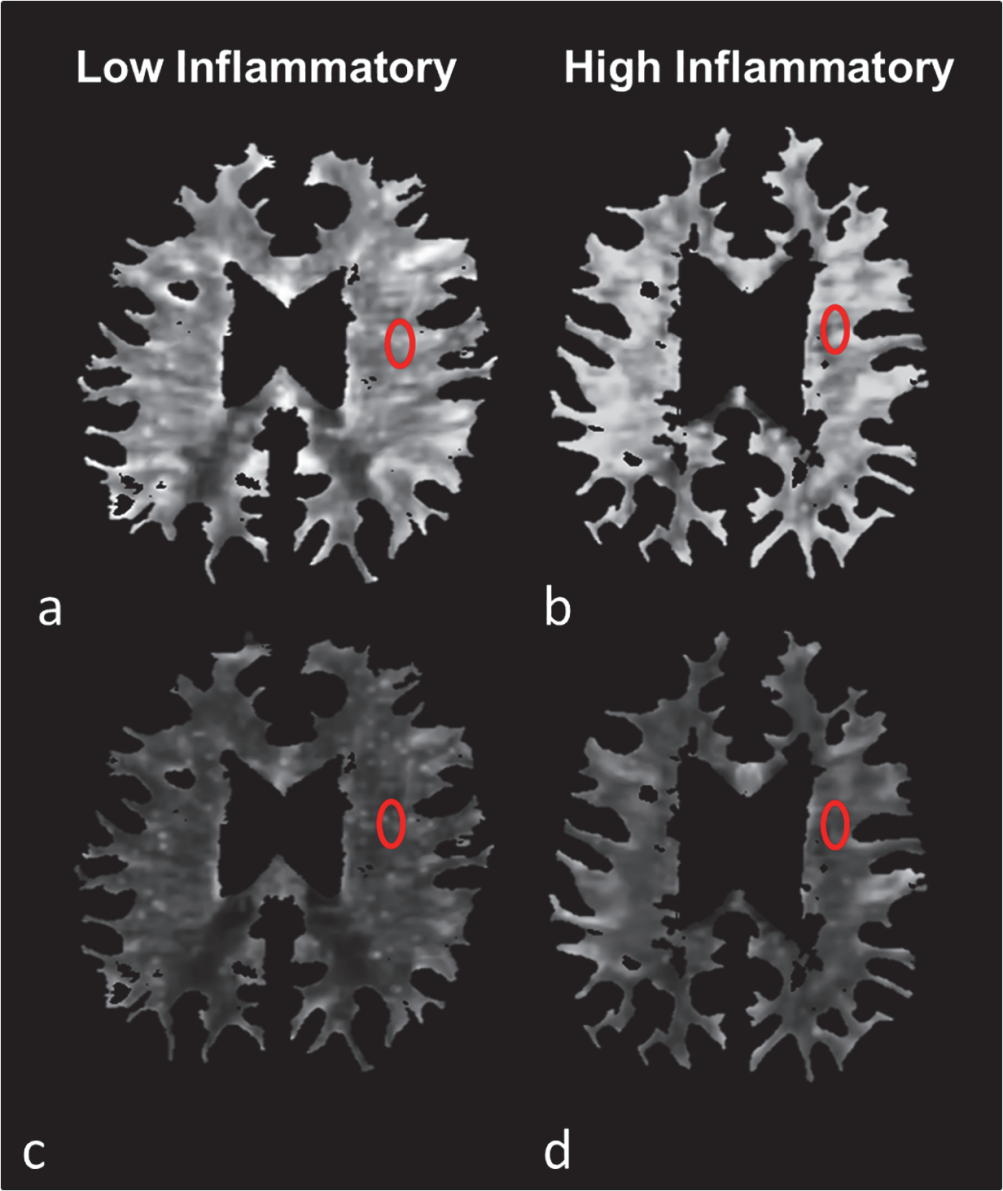
Segmentation results of white matter CBV (a,b) and CBF (c,d) maps with excluded lesions (NAWM) in a single low inflammatory and high inflammatory patient. Exemplary regions of interest drawn in the left frontal white matter of each individual map show elevated mean CBV (9.61 ± 1.35 vs 7.22 ± 0.77 ml/100g) and CBF (46.47 ±6.93 vs. 39.42 ± 6.32 ml/100g/min) in high inflammatory patients.

To acquire NAWM segmentations, CBV and CBF maps were masked with WM segmentation results excluding the T2 lesions (**Fig. 1**). To exclude pixels with low probability for WM from statistical analysis, the WM maps were tresholded at a value of 0.70 and the lesion maps were dilated (by 2 voxels). Normalized histograms with 256 bins were created from these images. From each histogram the mean value was extracted.

### Statistical analysis

Descriptive statistics are presented as absolute frequency or mean ± standard deviation [SD] or range (min-max). To examine differences between HI and LI group demographic baseline variables were compared using t-test or chi-squared test (whichever was appropriate). Time effects in imaging parameters were analyzed using mixed models with patient as random factor adjusting for the cluster structure in the data and including time as an independent factor. Due to asymmetric distributions of T2-LV, T1-LV and Gd-LV logarithms were used as dependent variables. In case of original scaled variables effect sizes are described as marginal means with 95%-confidence intervals (CI) and in case of log-transformed variables as percentage change (with 95%-CI).

To determine the correlation between imaging parameters and the two outcome variables of interest (CBV and CBF) we also calculated mixed models with patient as random factor. Independent variables comprised group (LI vs HI), imaging parameters (T2-LV, T1-LV, Gd-LV, GMV and WMV), time and EDSS and, moreover, age and gender as adjusting variables. Parameter estimates and marginal means with 95%-confidence intervals (CI) are presented. A p-value of <0.05 was considered to be statistically significant. Statistical analysis was performed with STATA 13.1 (STATA Corporation).

## Results

Thirteen patients (39%) were classified as high inflammatory according to their nGd-L count. There was no significant difference between the HI and LI group regarding demographic and imaging parameters except EDSS (p = 0.036), T2-LV (p = 0.019) and Gd-LV (p<0.001) (**Table 1).**

**Table 1:** Demographics, clinical and conventional MRI data obtained from the high inflammatory and low inflammatory patient group.

	high inflammatory group	low inflammatory group	p-value
**Age** (years, range)	35.6 (18–49)	39.0 (18–56)	0.417
**Gender** (**F/M**)	10/3	16/4	0.833
**DD** (year, ± SD)	5.44 ± 5.00	3.47 ± 4.69	0.246
**EDSS** (median and range)	1.58 (1–2.5)	2.03 (1–3)	**0.036**
**T2-LV** (mL mean ± SD)	5.47± 3.86	3.07 ± 3.21	**0.019**
**T1-LV** (mL mean ± SD)	0.46±0.69	0.92 ± 2.00	0.228
**Gd-LV** (mL mean ± SD)	0.15±0.18	0.02±0.05	**<0.001**
**GMV** (mL mean ± SD)	830.72± 49.76	831.93 ± 47.66	0.965
**WMV** (mL mean ± SD)	730.17± 24.20	727.92 ± 33.88	0.843

MRI derived data represent mean values from all acquired MRI examinations for both patient groups.

Abbreviations: SD = Standard deviation, DD = disease duration (years), EDSS = Expanded Disability Status Scale, T2-LV = T2 hyperintense lesion volume, T1-LV = T1 hypointense lesion volume, Gd-LV = contrast enhancing lesion volume, GMV = gray matter volume, WMV = white matter volume

Of all imaging parameters we found a significant change over time, analogous in both groups, in Gd-LV (overall p<0.001; 60.9% decrease from MRI-1 to MRI-2, 95%-CI [24.1;79.8]; p = 0.005). Longitudinal data for separate MRI timepoints is presented in a supplemental table (**S1 Table**).

### Extent of NAWM pathology measured by DSC-PWI

**Fig 2:**
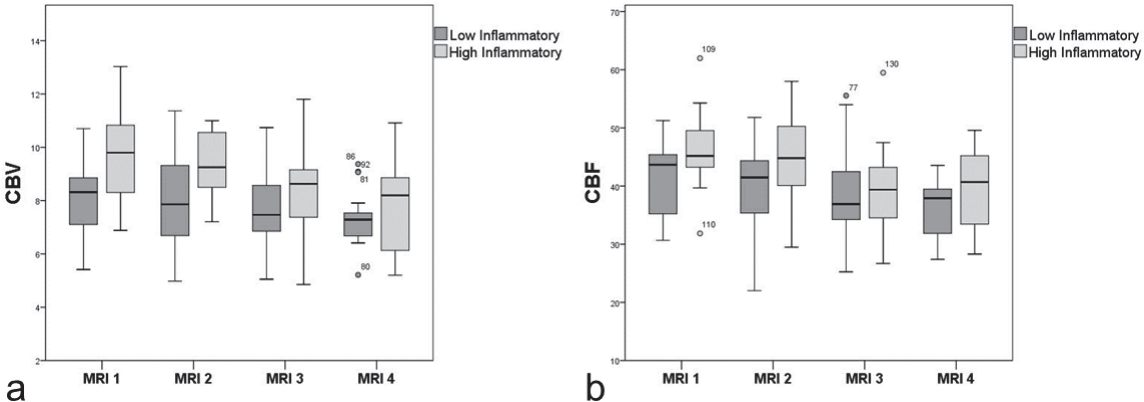
Temporal evolution of perfusion parameters in NAWM comparing high inflammatory with low inflammatory patients: a) CBV, b) CBF.

Compared with LI patients, the HI group showed significantly higher CBV (9.1 vs 7.6 ml/100g, p<0.001) and CBF (43.7 vs 38.7 ml/100g/min, p = 0.014). CBV and CBF in NAWM decreased significantly over time in both groups (CBV: overall p = 0.001, MRI2 vs MRI-1: p = 0.535; MRI-3 vs MRI-1: β = −0.6 [−1.1; −0.1], p = 0.011; MRI-4 vs MRI-1: β = −0.9 [−1.4; −0.4], p<0.001) and CBF: overall p = 0.002, MRI-2 vs MRI-1: p = 0.463; MRI-3 vs MRI-1: β = −3.5 [−6.3; −0.8], p = 0.012; MRI-4 vs MRI-1: β = −5.1 [−7.9; −2.2], p<0.001). We did not find a significant group-by-time interaction for CBV (p = 0.215) or CBF (p = 0.445) displaying a analogous decrease over time of both variables in the LI and HI group. **Fig. 2** demonstrates the temporal changes of CBV and CBF in both patient groups.

### Relationship of WM injury and measures of lesion and brain volume

For CBF, only T1-LV showed a significant correlation (β = −1.5 [−2.6; −0.3]; p = 0.013). Neither T2-LV, nor Gd-LV or atrophy measurements revealed significant correlations. We did not find any significant correlations of imaging parameters in the model for CBV (**Table 2)**.

**Table 2:** Mixed model coefficients for correlation of perfusion parameters in NAWM with WM and atrophy measurements (all models adjusted for age, gender and time).

	CBV NAWM		CBF NAWM	
	β [95%-CI]	p	β [95%-CI]	p
**Group (ref = LI)**	1.45 [0.56;2.35]	**0.001**	4.97 [1.01;8.93]	**0.014**
**T2-LV**	−0.03 [−0.12;0.07]	0.595	−0.09 [−0.58;0.40]	0.715
**T1-LV**	−0.23 [−0.48;0.02]	0.070	−1.48 [−2.64;-0.31]	**0.013**
**Gd-LV**	0.51 [−1.52;2.54]	0.621	4.10 [−6.58;14.78]	0.452
**WMV**	0.01 [−0.00;0.02]	0.115	0.03 [−0.02;0.08]	0.182
**GMV**	−0.00 [−0.01;0.01]	0.952	−0.02 [−0.06;0.03]	0.488
**EDSS**	0.88 [0.02;1.75]	**0.046**	5.20 [1.39;9.02]	**0.008**

Abbreviations: CBV = cerebral blood volume, CBF = cerebral blood flow, CI = confidence interval, T2-LV = T2 hyperintense lesion volume, T1-LV = T1 hypointense lesion volume, Gd-LV = contrast enhancing lesion volume, WMV = white matter volume, GMV = gray matter volume, EDSS = Expanded Disability Status Scale

### Relationship of WM pathology and clinical parameters

For both CBV and CBF, mixed models revealed a significant association of EDSS with CBV (β = 0.9 [0.0;1.8]; p = 0.046) and CBF (β = 5.2 [1.4;9.0]; p = 0.008) (**Table 2**).

## Discussion

We studied DSC-PWI in NAWM of RR-MS patients comparing a high- with a low-inflammatory cohort. This work provides evidence that a high inflammatory lesion load is associated with increased CBV and CBF in NAWM, confirming the original hypothesis and underlining the relevance of blood-flow as a surrogate for inflammation.

Several publications have described hyperperfusion in WM of MS patients. Studies focusing on lesions reported elevated perfusion parameters in contrast enhancing lesions compared to contralateral NAWM [14,23] as well as increased regional perfusion to occur several weeks prior to contrast enhancement of MS lesions [13]. Papadaki et al [24] identified elevated CBV in NAWM of MS patients with a clinically isolated syndrome supporting the idea of hyperperfusion in early disease while latent hypoperfusion in late disease associated with cognitive deficits. Ingrisch et al [25] reported a significantly increased perfusion in contrast enhancing lesions compared to relative low perfusion values of NAWM. The authors attribute their findings to vasodilatation due to inflammation. As we did not assess perfusion parameters in Gd-L, the relationship of Gd-L to NAWM perfusion in a high inflammatory cohort could not be confirmed in this study. Rashid et al. [11] described elevated CBV/CBF in NAWM of RR-MS only measurable in untreated patients utilizing arterial spin-labeling (ASL) MRI. Further evidence of the potential role of inflammation in WM in MS is provided by PET studies that showed increased activity in gadolinium enhancing lesions and in NAWM [26].

Altogether, these findings are well in line with our results. We found a strong relationship between inflammation seen in conventional MRI and elevated perfusion based on the fact that the number and volume of contrast enhancing lesions was the only contributor to NAWM perfusion increase. The linear relationship between CBV in NAWM and Gd-L/nGd-L corroborates our assumption of direct relationship of contrast enhancing lesions and global metabolic activity.

This is also in agreement with the evolution of MS-related pathology. The first inflammatory phase is characterized by increased BBB permeability, extravasations of lymphocytes, and activation/proliferation of macrophages [27]. It is reasonable that this stage is associated with an increased metabolism due to increase in vasodilatory nitric oxide (NO) and glutamate [28]. Effective vasodilators such as NO and substance P presumably originate from brain inflammation [29,30]. Substance P producing immune reactive cells were identified at the edge of active MS lesions, suggesting that SP may play a role in MS lesion development [30]. Global arteriolar vasodilation could represent a physiologic response to increased oxygen demand, which in the context of MS could be secondary to increased inflammatory- and glial-cell activity. Second, new vessel formation, along with increased vascular endothelial growth factor expression, have been shown early in the development of MS lesions [31,32]. CBV has been proven to be a sensitive marker of angiogenesis, with significant correlations between the increase of CBV and CBF values and microvessel density [33]. Notably, the arrangement of angiogenic vessels in MS appears orderly whereas tumor angiogenesis is chaotic [34] suggesting a reparative response to injury [31].

Although no immunohistochemical data is currently available, it is possible that the elevated CBV/CBF values in the NAWM in the HI cohort may be indicative of angiogenesis occurring additionally to inflammation-induced vasodilatation [24].

As such, the increased CBV/CBF values in NAWM could reflect inflammation (before a local BBB-breakdown) that remains undetectable by conventional post-contrast MRI but is coexistent to focal BBB-breakdown.

Most studies reporting hypoperfusion in NAWM also identified a correlation of perfusion values with neuropsychological deficits as fatigue [35] and cognitive deficits [36]. Those clinical manifestations of MS are more likely the consequence of neurodegeneration rather than acute inflammation [37]. Other studies reporting decreased CBV and CBF in NAWM do not comment on the absolute number of contrast enhancing lesions in their patient groups [9,10,14,38]. Therefore this data is hardly comparable to our HI cohort. In summary, the abovementioned literature provides strong evidence for decreased perfusion in NAWM and lesions at least in some stages of the disease.

The pathological substrate of hypoperfusion in MS is usually described as a primary vascular pathology rather than decreased metabolic demand. Microvascular damage such as small venous thrombosis and vein wall hyalinization and intravascular fibrin deposition have been observed in MS lesions [5]. Cytotoxic T cells recognize their antigen on endothelial cells and activate a clotting cascade which may lead to thrombosis. Toxic inflammatory mediators contribute to tissue hypoxia through mitochondrial injury [39,40]. Furthermore, inflammatory edema may impair microcirculation through focal tissue swelling [41].

In contrast to our data Juurlink et al [42] discuss a possible role of hypoperfusion even in the formation of acute contrast enhancing lesions possibly due to decreased arterial supply or restricted venous return. A potential concern with this idea is that hypoperfusion and subsequent oligodendrocyte as well as myelin damage as well as expression of pro-inflammatory genes in the context of ischemic stroke do not result in an MS-like immune attack on myelin. The role of venous drainage alteration in MS in the form of microvascular damage such as small venous thrombosis, vein wall hyalinization and intravascular fibrin deposition has been observed in MS lesions at histological examination [5,6], while macrovascular venous obstruction in MS remains a controversial topic [43].

Taken together with our data, we assume that our data presents evidence for increased perfusion in a highly inflammatory period of the disease with evidence of BBB breakdown while other stages of MS seem to be associated with decreased perfusion in NAWM.

It remains difficult to explain the linear decrease of perfusion values over time as the effect is similar for both the HI and LI group. Cramer et al [44] investigated the integrity of the BBB and describe a gradual decrease of permeability after an MS relapse. This data could provide a possible explanation of a return to “normal” or even to a hypoperfusion state after an incidence of strong inflammation. Although this effect is significant even in the LI group, we do see a significant amount of contrast enhancing lesions even in this group. To examine the possibility of receding global inflammation and perfusion values returning to “normal” state a longer follow up period and a healthy control group would be needed. The positive correlation of EDSS and elevated CBV and CBF values substantiate the clinical impact of our data. This effect may be partly driven by the high contrast enhancing lesion load in our cohort, nevertheless, the lack of correlation of Gd-LV with elevated CBV and CBF suggests an effect of elevated perfusion on the clinical status independent from the contrast enhancing lesion load. Those results are in contrast to data published by Adhya at al. [9] who reported a correlation between regional hypoperfusion and EDSS and other studies describing association of hypoperfusion with neuropsychological symptoms such as cognitive impairment [36], fatigue [45] and memory dysfunction [46]. Altogether, those results may be explained by inflammatory related vasodilatation in the active inflammatory stage with decreased perfusion in later stages of the disease without active inflammation.

Neither CBF nor CBV values correlate with disease duration and T2-LV, suggesting that WM dysfunction may occur at any time of the disease and cannot be explained by the effects of WM lesions.

This study has some limitations. First, we considered global instead of local perfusion changes. However our approach allowed a straightforward testing for the determination of associated factors leading to increased NAWM perfusion in MS.

Additionally, the histogram analysis revealed a unimodal distribution of CBV/CBF suggesting a relatively homogenous scattering of perfusion values. Furthermore, there is a still an ongoing debate which imaging technique should be used in MS. PET still represents the best method for absolute perfusion quantification in the brain [26], but it requires a radioactive tracer and is not widely available. Cerebral perfusion can be investigated using various MR imaging techniques, such as fMRI, ASL, and DSC-PWI. While the endogenous contrast agents used by fMRI and ASL are less invasive, DSC-PWI imaging is more frequently performed in the clinical setting and offers a better signal-to-noise ratio. Absolute perfusion values were used in this study since measurements of perfusion parameters relative to the contralateral white matter may be erroneous, as it is known that the (contralateral) NAWM is abnormal in MS [8].

In theory, deconvolution-based perfusion analysis is capable to correct for differences resulting from the cardiac output leading to absolute quantitative perfusion measurements. However, these methods require a previous definition of an arterial input function, whereas a recent study by Paling et al. [12] has demonstrated significantly prolonged cerebral arterial bolus arrival times in MS patients, which makes the deconvolution-based perfusion analysis in MS patients questionable. The fact that the simple fitting of the local density random walk model does not correct for the arterial input may explain the relatively high CBV values in our cohort. Although this model provides only semi-quantitative perfusion values that are not directly comparable to other methods, it provides robust and consistent parameters that are well comparable inside our cohort as great care was taken to keep the contrast injection protocol equal for all patients. Another limitation of this study is the lack of a healthy control group. Although the CBV and CBF values in both examined MS groups presented in this study are definitely increased compared to previously published data in healthy control groups [9,10], a healthy cohort would have increased the statistical power of this study. However, the application of gadoterate dimeglumine to acquire DSC-PWI in a healthy control group always presents an unnecessary risk and is therefore unlikely to be approved by our local ethics committee.

### Conclusions

This study shows that the NAWM of high inflammatory MS patients is characterized by concomitant increase of CBV/CBF values indicative of a global vascular pathology. This may have important clinical implications in both disease pathogenesis and development of experimental therapies in MS. PWI could serve as a new surrogate marker for global inflammation in clinical studies.

## Supporting Information

S1 Table Cross-sectional MRI data separated for the high and low inflammatory group and the different MRI acquisitions. (DOCX)

## References

[1] Barkhof F. MRI in multiple sclerosis: correlation with expanded disability status scale (EDSS). Mult Scler 1999;5: 283–286. 10467389 10.1177/135245859900500415

[2] Zeis T, Graumann U, Reynolds R, Schaeren-Wiemers N. Normal-appearing white matter in multiple sclerosis is in a subtle balance between inflammation and neuroprotection. Brain 2008;131: 288–303. 18056737 10.1093/brain/awm291

[3] Raz E, Cercignani M, Sbardella E, Totaro P, Pozzilli C, et al Clinically isolated syndrome suggestive of multiple sclerosis: voxelwise regional investigation of white and gray matter. Radiology 2010;254: 227–234. 10.1148/radiol.2541090817 20019140

[4] Putnam TJ. The pathogenesis of multiple sclerosis: a possible vascular factor New England Journal of Medicine 1933

[5] Wakefield AJ, More LJ, Difford J, McLaughlin JE. Immunohistochemical study of vascular injury in acute multiple sclerosis. J Clin Pathol 1994;47: 129–133. 8132826 10.1136/jcp.47.2.129 PMC501826

[6] Adams CW, Poston RN, Buk SJ, Sidhu YS, Vipond H. Inflammatory vasculitis in multiple sclerosis. J Neurol Sci 1985;69: 269–283. 4031947 10.1016/0022-510x(85)90139-x

[7] Soon D, Tozer DJ, Altmann DR, Tofts PS, Miller DH. Quantification of subtle blood-brain barrier disruption in non-enhancing lesions in multiple sclerosis: a study of disease and lesion subtypes. Mult Scler 2007;13: 884–894. 17468443 10.1177/1352458507076970

[8] Law M, Saindane AM, Ge Y, Babb JS, Johnson G, et al Microvascular Abnormality in Relapsing-Remitting Multiple Sclerosis: Perfusion MR Imaging Findings in Normal-appearing White Matter. Radiology 2004;231: 645–652. 15163806 10.1148/radiol.2313030996

[9] Adhya S, Johnson G, Herbert J, Jaggi H, Babb JS, et al Pattern of hemodynamic impairment in multiple sclerosis: Dynamic susceptibility contrast perfusion MR imaging at 3.0 T. Neuroimage 2006;33: 1029–1035. 16996280 10.1016/j.neuroimage.2006.08.008 PMC1752216

[10] Varga AW, Johnson G, Babb JS, Herbert J, Grossman RI, et al White matter hemodynamic abnormalities precede sub-cortical gray matter changes in multiple sclerosis. J Neurol Sci 2009;282: 28–33. 10.1016/j.jns.2008.12.036 19181347 PMC2737614

[11] Rashid W, Parkes LM, Ingle GT, Chard DT, Toosy AT, et al Abnormalities of cerebral perfusion in multiple sclerosis. J Neurol Neurosurg Psychiatr 2004;75: 1288–1293. 15314117 10.1136/jnnp.2003.026021 PMC1739228

[12] Paling D, Thade Petersen E, Tozer DJ, Altmann DR, Wheeler-Kingshott CAM, et al Cerebral arterial bolus arrival time is prolonged in multiple sclerosis and associated with disability. J Cereb Blood Flow Metab 2014;34: 34–42. 10.1038/jcbfm.2013.161 24045400 PMC3887342

[13] Wuerfel J, Bellmann-Strobl J, Brunecker P, Aktas O, McFarland H, et al Changes in cerebral perfusion precede plaque formation in multiple sclerosis: a longitudinal perfusion MRI study. Brain 2004,127: 111–119. 14570816 10.1093/brain/awh007

[14] Ge Y, Law M, Johnson G, Herbert J, Babb JS, et al Dynamic susceptibility contrast perfusion MR imaging of multiple sclerosis lesions: characterizing hemodynamic impairment and inflammatory activity. Am J Neuroradiol 2005;26: 1539–1547. 15956527 PMC8149080

[15] Polman CH, Reingold SC, Banwell B, Clanet M, Cohen JA, et al Diagnostic criteria for multiple sclerosis: 2010 revisions to the McDonald criteria. Ann Neurol 2011;69: 292–302. 10.1002/ana.22366 21387374 PMC3084507

[16] Giovannoni G, Radue E-W, Havrdova E, Riester K, Greenberg S, et al Effect of daclizumab high-yield process in patients with highly active relapsing-remitting multiple sclerosis. J Neurol 2014;261: 316–323. 10.1007/s00415-013-7196-4 24375015 PMC3915085

[17] Kurtzke JF. Rating neurologic impairment in multiple sclerosis: an expanded disability status scale (EDSS). Neurology 1983;33: 1444–1452. 6685237 10.1212/wnl.33.11.1444

[18] Smith SM, Zhang Y, Jenkinson M, Chen J, Matthews PM, et al Accurate, robust, and automated longitudinal and cross-sectional brain change analysis. Neuroimage 2002;17: 479–489. 12482100 10.1006/nimg.2002.1040

[19] Battaglini M, Jenkinson M, De Stefano N. Evaluating and reducing the impact of white matter lesions on brain volume measurements. Human brain mapping 2012;33: 2062–2071. 10.1002/hbm.21344 21882300 PMC6870255

[20] Forkert ND, Cheng B, Kemmling A, Thomalla G, Fiehler J. ANTONIA Perfusion and Stroke. A Software Tool for the Multi-purpose Analysis of MR Perfusion-weighted Datasets and Quantitative Ischemic Stroke Assessment. Methods Inf Med 2014;53(6): 469–481. 10.3414/ME14-01-0007 25301390

[21] Li X, Tian J, Millard RK. Erroneous and inappropriate use of gamma fits to tracer-dilution curves in magnetic resonance imaging and nuclear medicine. Magn Reson Imaging 2003;21: 1095–1096. 14684217 10.1016/s0730-725x(03)00205-4

[22] Greve DN, Fischl B. Accurate and robust brain image alignment using boundary-based registration. Neuroimage 2009;48: 63–72. 10.1016/j.neuroimage.2009.06.060 19573611 PMC2733527

[23] Haselhorst RR, Kappos LL, Bilecen DD, Scheffler KK, Möri DD, et al Dynamic susceptibility contrast MR imaging of plaque development in multiple sclerosis: application of an extended blood-brain barrier leakage correction. Journal of magnetic resonance imaging: JMRI 2000;11: 495–505. 10813859 10.1002/(sici)1522-2586(200005)11:5<495::aid-jmri5>3.0.co;2-s

[24] Papadaki EZ, Mastorodemos VC, Amanakis EZ, Tsekouras KC, Papadakis AE, et al White matter and deep gray matter hemodynamic changes in multiple sclerosis patients with clinically isolated syndrome. Magnetic resonance in medicine: official journal of the Society of Magnetic Resonance in Medicine / Society of Magnetic Resonance in Medicine 2012;68: 1932–1942. 10.1002/mrm.24194 22367604

[25] Ingrisch MM, Sourbron SS, Morhard DD, Ertl-Wagner BB, Kümpfel TT, et al Quantification of perfusion and permeability in multiple sclerosis: dynamic contrast-enhanced MRI in 3D at 3T. Investigative radiology 2012;47: 252–258. 10.1097/RLI.0b013e31823bfc97 22373532

[26] Banati RB, Newcombe J, Gunn RN, Cagnin A, Turkheimer F, et al The peripheral benzodiazepine binding site in the brain in multiple sclerosis: quantitative in vivo imaging of microglia as a measure of disease activity. Brain 2000;123 (Pt 11): 2321–2337. 11050032 10.1093/brain/123.11.2321

[27] Lucchinetti CF, Parisi J, Bruck W. The pathology of multiple sclerosis. Neurologic clinics 2005;23: 77–105. 15661089 10.1016/j.ncl.2004.09.002

[28] Haider L, Fischer MT, Frischer JM, Bauer J, Höftberger R, et al Oxidative damage in multiple sclerosis lesions. Brain 2011;134: 1914–1924. 10.1093/brain/awr128 21653539 PMC3122372

[29] Hartung T, Sauer A, Wendel A. Testing of immunomodulatory properties in vitro. Dev Biol Stand 1996;86: 85–96. 8785996

[30] Kostyk SK, Kowall NW, Hauser SL. Substance P immunoreactive astrocytes are present in multiple sclerosis plaques. Brain Res 1989;504: 284–288. 2480834 10.1016/0006-8993(89)91369-3

[31] Holley JE, Newcombe J, Whatmore JL, Gutowski NJ. Increased blood vessel density and endothelial cell proliferation in multiple sclerosis cerebral white matter. Neurosci Lett 2010;470: 65–70. 10.1016/j.neulet.2009.12.059 20036712

[32] Roscoe WA, Welsh ME, Carter DE, Karlik SJ. VEGF and angiogenesis in acute and chronic MOG((35–55)) peptide induced EAE. J Neuroimmunol 2009;209: 6–15. 10.1016/j.jneuroim.2009.01.009 19233483

[33] Pathak AP, Schmainda KM, Ward BD, Linderman JR, Rebro KJ, et al MR-derived cerebral blood volume maps: issues regarding histological validation and assessment of tumor angiogenesis. Magnetic resonance in medicine: official journal of the Society of Magnetic Resonance in Medicine 2001;46: 735–747. 11590650 10.1002/mrm.1252

[34] Jain RK. Tumor angiogenesis and accessibility: role of vascular endothelial growth factor. Semin Oncol 2002;29: 3–9. 12516032 10.1053/sonc.2002.37265

[35] Inglese M, Park S-J, Johnson G, Babb JS, Miles L, et al Deep gray matter perfusion in multiple sclerosis: dynamic susceptibility contrast perfusion magnetic resonance imaging at 3 T. Arch Neurol 2007;64: 196–202. 17296835 10.1001/archneur.64.2.196

[36] Inglese M, Adhya S, Johnson G, Babb JS, Miles L, et al Perfusion magnetic resonance imaging correlates of neuropsychological impairment in multiple sclerosis. J Cereb Blood Flow Metab 2007;28: 164–171. 17473851 10.1038/sj.jcbfm.9600504 PMC2596621

[37] Filippi M, Rocca MA, Benedict RHB, DeLuca J, Geurts JJG, et al The contribution of MRI in assessing cognitive impairment in multiple sclerosis. Neurology 2010;75: 2121–2128. 10.1212/WNL.0b013e318200d768 21135387 PMC3385423

[38] Law M, Saindane AM, Ge Y, Babb JS, Johnson G, et al Microvascular Abnormality in Relapsing-Remitting Multiple Sclerosis: Perfusion MR Imaging Findings in Normal-appearing White Matter. Radiology 2004;231: 645–652. 15163806 10.1148/radiol.2313030996

[39] Kalman B, Leist TP. A mitochondrial component of neurodegeneration in multiple sclerosis. Neuromolecular Med 2003;3: 147–158. 12835510 10.1385/NMM:3:3:147

[40] Heales SJ, Bolaños JP, Stewart VC, Brookes PS, Land JM, et al Nitric oxide, mitochondria and neurological disease. Biochim Biophys Acta 1999;1410: 215–228. 10076028 10.1016/s0005-2728(98)00168-6

[41] Lassmann H. Hypoxia-like tissue injury as a component of multiple sclerosis lesions. J Neurol Sci 2003;206: 187–191. 12559509 10.1016/S0022-510X(02)00421-5 PMC7130136

[42] Juurlink BHJ. The evidence for hypoperfusion as a factor in multiple sclerosis lesion development. Mult Scler Int 2013;2013:598093 10.1155/2013/598093 23691321 PMC3649777

[43] Khan O, Filippi M, Freedman MS, Barkhof F, Dore-Duffy P, et al Chronic Cerebrospinal Venous Insufficiency and Multiple Sclerosis. Ann Neurol 2010;67: 286–290. 10.1002/ana.22001 20373339

[44] Cramer SP, Simonsen H, Frederiksen JL, Rostrup E, Larsson HBW. Abnormal blood-brain barrier permeability in normal appearing white matter in multiple sclerosis investigated by MRI. Neuroimage Clin 2014;4: 182–189. 10.1016/j.nicl.2013.12.001 24371801 PMC3872721

[45] Inglese M, Park S-J, Johnson G, Babb JS, Miles L, et al Deep gray matter perfusion in multiple sclerosis: dynamic susceptibility contrast perfusion magnetic resonance imaging at 3 T. Arch Neurol 2007;64: 196–202. 17296835 10.1001/archneur.64.2.196

[46] Papadaki EZ, Simos PG, Panou T, Mastorodemos VC, Maris TG, et al Hemodynamic evidence linking cognitive deficits in clinically isolated syndrome to regional brain inflammation. Eur J Neurol 2014;21: 499–505. 10.1111/ene.12338 24373026

