# Correction: Increased Perfusion in Normal Appearing White Matter in High Inflammatory Multiple Sclerosis Patients

**DOI:** 10.1371/journal.pone.0142464

**Published:** 2015-11-04

**Authors:** Maxim Bester, Nils Daniel Forkert, Jan Patrick Stellmann, Klarissa Stürner, Lilian Aly, Anna Drabik, Kim Lea Young, Christoph Heesen, Jens Fiehler, Susanne Siemonsen

Dr. Klarissa Stürner should be included in the author byline. She should be listed as the fourth author, and her affiliation is Institute of Neuroimmunology and Multiple Sclerosis Research, UMC Hamburg, Hamburg, Germany. The contributions of the author is as follows: contributed reagents/materials/analysis tools, handling of patients and clinical data.

The correct citation is: Bester M, Forkert ND, Stellmann JP, Stürner K, Aly L, Drabik A, et al. (2015) Increased Perfusion in Normal Appearing White Matter in High Inflammatory Multiple Sclerosis Patients. PLoS ONE 10(3): e0119356. doi:10.1371/journal.pone.0119356
