# Supplementary material for: Increased Perfusion in Normal Appearing White Matter in High Inflammatory Multiple Sclerosis Patients
Source: PLoS One. 2015 Mar 16;10(3):e0119356. doi: 10.1371/journal.pone.0119356 (PMC4361628; doi:10.1371/journal.pone.0119356)
Supplement: S1 Table — (DOCX) [file pone.0119356.s001.docx]

**Supplemental Table S1:** Crossectional MRI data separated for the high and low inflammatory group and the different MRI acquisitions

|  | MRI-1 | | | | MRI-2 | | | | MRI-3 | | | | MRI-4 | | | |
| --- | --- | --- | --- | --- | --- | --- | --- | --- | --- | --- | --- | --- | --- | --- | --- | --- |
|  | LI | | HI | | LI | | HI | | LI | | HI | | LI | | HI | |
|  | mean | SD | mean | SD | mean | SD | mean | SD | mean | SD | mean | SD | mean | SD | mean | SD |
| CBF **(**mL/100g mean ± SD**)** | 38.63 | 5.86 | 43.29 | 6.16 | 38.63 | 5.86 | 43.29 | 6.16 | 38.63 | 5.86 | 43.29 | 6.16 | 38.63 | 5.86 | 43.29 | 6.16 |
| CBV **(**mL/100g/min mean ± SD**)** | 7.66 | 1.23 | 9.00 | 1.42 | 7.66 | 1.23 | 9.00 | 1.42 | 7.66 | 1.23 | 9.00 | 1.42 | 7.66 | 1.23 | 9.00 | 1.42 |
| T2-LV **(**mL mean ± SD**)** | 3.09 | 2.69 | 4.96 | 3.94 | 3.27 | 3.19 | 5.05 | 3.61 | 2.46 | 2.32 | 6.44 | 4.40 | 3.47 | 4.47 | 5.42 | 3.70 |
| T1-LV **(**mL mean ± SD**)** | 0.84 | 1.79 | 0.49 | 0.82 | .87 | 1.81 | 0.37 | 0.60 | 1.13 | 2.55 | 0.47 | 0.64 | 0.83 | 1.89 | 0.53 | 0.75 |
| Gd-LV **(**mL mean ± SD**)** | 0.02 | 0.03 | 0.16 | 0.16 | 0.01 | 0.04 | 0.11 | 0.15 | 0.04 | 0.09 | 0.23 | 0.25 | 0.03 | 0.05 | 0.11 | 0.09 |
| Gd-L **(**mean ± SD**)** | 0.70 | 0.97 | 4.08 | 3.09 | 0.10 | 0.31 | 3.92 | 5.12 | 0.45 | 0.51 | 5.15 | 4.52 | 0.84 | 1.11 | 4.00 | 3.89 |
| nGd-L **(**mean ± SD**)** | 0.70 | 0.97 | 4.08 | 3.09 | 0.05 | 0.22 | 2.85 | 3.41 | 0.47 | 0.49 | 4.38 | 3.38 | 0.63 | 0.89 | 3.15 | 3.99 |
| GMV **(**mL mean ± SD**)** | 838.71 | 50.18 | 831.17 | 47.02 | 836.04 | 50.52 | 841.76 | 55.95 | 829.27 | 48.31 | 825.95 | 49.70 | 823.12 | 43.25 | 823.44 | 49.82 |
| WMV **(**mL mean ± SD**)** | 725.49 | 36.11 | 731.53 | 29.71 | 723.31 | 30.97 | 732.85 | 25.95 | 730.76 | 34.46 | 730.37 | 22.55 | 732.49 | 35.70 | 725.71 | 20.11 |

Abbreviations: SD= Standard deviation, CBF= Cerebral blood flow, CBV = Cerebral blood volume, T2-LV = T2 hyperintense lesion volume, T1-LV = T1 hypointense lesion volume, Gd-LV = contrast enhancing lesion volume, nGd-L= number of new gadolinium enhancing lesions, Gd-L= total number of gadolinium enhancing lesions, GMV = gray matter volume, WMV = white matter volume
